# The indispensable role of disaster preparedness in venous thromboembolism prevention: Insights from Japan

**DOI:** 10.1016/j.jvsv.2025.102313

**Published:** 2026-01-08

**Authors:** Rashad A. Bishara

**Affiliations:** General Organization for Teaching Hospitals and Institutes, Cairo, Egypt

The article "Disaster-associated venous thromboembolism countermeasures in Japan: Insights from past major earthquakes" by Kamada et al^1^ provides a critical review of disaster-associated venous thromboembolism (VTE) and the exemplary efforts undertaken by the Japanese Society of Phlebology to prevent this important public health risk. In a time where natural disasters and wars are increasing in frequency, this review's impact goes far beyond the borders of Japan.

The authors emphasize how the unique environment of a postdisaster setting, namely, immobility, dehydration, and stress, leads to a dramatic increase in cardiovascular events and in VTE incidence. The statistics showing a deep vein thrombosis (DVT) incidence of 10% to 30% after earthquakes are particularly striking. The manuscript's discussion of the Japanese Disaster Countermeasure Committee provides a good example of a multidisciplinary approach to prevent the potentially fatal complication of VTE. They nicely demonstrated that measures such as public education, DVT screening, the use of compression stockings, and the use of cardboard beds in shelters are practical and highly effective.

The combination of awareness campaigns among the public and health care professionals, as well as the collaboration between various medical assistance teams, may serve as a model for other countries to follow. The Japan-Surveillance in Post Extreme Emergencies and Disasters disaster medical reporting system is also a demonstration of how a commitment to data collection and surveillance is essential for refining disaster countermeasures.

Although this article describes the VTE high-risk conditions after earthquakes, the principles outlined apply to all disasters, such as hurricanes, floods, and severe wildfires, which necessitate the evacuation of residents into shelter areas. The insights are particularly relevant for war-torn regions where displaced populations face similar challenges in refugee camps and shelters.^2^^,^^3^

The authors described a simplified protocol for ultrasound screening for DVT, which takes into account the limited space and time during disaster times. The individuals considered at high risk were examined while standing; the examination focused on the calf and popliteal veins. This is a quick and effective method to detect VTE. The authors cite that "more than 90% of massive [pulmonary embolism] originates in the deep veins of the lower legs based on autopsy findings."^4^

The authors advocate for the use of compression stockings in these settings as a preventive measure for VTE, based on their experience of reducing perioperative pulmonary embolism by nearly half since the widespread adoption of early mobilization and compression stockings in hospitalized patients in Japan.^5^ This stands in contrast with some major Western guidelines, such as those from the American College of Chest Physicians, which have been more cautious owing to a lack of large-scale randomized controlled trials specific to these extreme, nonhospital settings.^6^

The article concludes by emphasizing the need for continued knowledge sharing. This is a call to action for the global vascular community. Japan's experience is remarkable in disaster countermeasures, and the integration of VTE prevention into our national disaster and mass casualty response protocols would be highly beneficial.

*The opinions or views expressed in this commentary are those of the authors and do not necessarily reflect the opinions or recommendations of the Journal of Vascular Surgery: Venous and Lymphatic Disorders or the Society for Vascular Surgery.*

## Funding

None.

## Disclosures

None.
